# 4-Chloro-*N*-(3-phenyl­allyl­idene)aniline

**DOI:** 10.1107/S1600536808039391

**Published:** 2008-11-29

**Authors:** Bing-Xiang Zhang

**Affiliations:** a Department of Chemistry, Taishan University, 271021 Taian, Shandong, People’s Republic of China

## Abstract

In the title mol­ecule, C_15_H_12_ClN, the C=N and C=C bond lengths are 1.273 (2) and 1.324 (2) Å, respectively. The two aromatic rings form a dihedral angle of 3.27 (3)°.

## Related literature

For a related structure, see Pu (2008 ▶). For general background, see: Garnovskii *et al.* (1993 ▶); Anderson *et al.* (1997 ▶); Musie *et al.* (2001 ▶); Paul *et al.* (2002 ▶). For bond-length data, see: Allen *et al.* (1987 ▶).
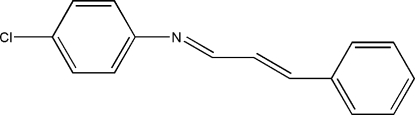


## Experimental

### 

#### Crystal data

C_15_H_12_ClN
*M* *_r_* = 241.71
Orthorhombic, 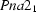

*a* = 7.7333 (7) Å
*b* = 5.5957 (5) Å
*c* = 29.383 (3) Å
*V* = 1271.5 (2) Å^3^
*Z* = 4
Mo *K*α radiation
μ = 0.28 mm^−1^
*T* = 295 (2) K
0.15 × 0.12 × 0.08 mm

#### Data collection

Bruker APEXII CCD area-detector diffractometer
Absorption correction: multi-scan (*SADABS*; Bruker, 2005 ▶) *T*_min_ = 0.960, *T*_max_ = 0.978
6043 measured reflections
2187 independent reflections
2042 reflections with *I* > 2σ(*I*)
*R*_int_ = 0.016

#### Refinement

*R*[*F* ^2^ > 2σ(*F* ^2^)] = 0.026
*wR*(*F* ^2^) = 0.066
*S* = 1.05
2187 reflections
154 parameters
H-atom parameters constrained
Δρ_max_ = 0.09 e Å^−3^
Δρ_min_ = −0.15 e Å^−3^
Absolute structure: Flack (1983 ▶), 1031 Friedel pairs
Flack parameter: 0.07 (5)

### 

Data collection: *APEX2* (Bruker, 2005 ▶); cell refinement: *APEX2*; data reduction: *SAINT* (Bruker, 2005 ▶); program(s) used to solve structure: *SHELXTL* (Sheldrick, 2008 ▶); program(s) used to refine structure: *SHELXTL*; molecular graphics: *SHELXTL*; software used to prepare material for publication: *SHELXTL*.

## Supplementary Material

Crystal structure: contains datablocks global, I. DOI: 10.1107/S1600536808039391/cv2477sup1.cif

Structure factors: contains datablocks I. DOI: 10.1107/S1600536808039391/cv2477Isup2.hkl

Additional supplementary materials:  crystallographic information; 3D view; checkCIF report

## Figures and Tables

**Fig. 1.**
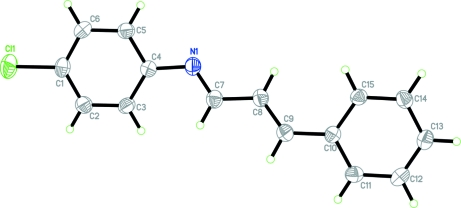
The molecular structure of (I), with displacement ellipsoids drawn at the 30% probability level.

## supplementary crystallographic information

### Comment

Schiff base compounds have been of great interest for many years. These compounds play an important role in the development of coordination chemistry related to catalysis and enzymatic reactions, magnetism and molecular architectures (Garnovskii *et al.*, 1993; Anderson *et al.*, 1997; Musie *et al.*, 2001; Paul *et al.*, 2002; Pu, 2008). In order to search for new Schiff-bases with higher bioactivity, the title compound (I) was synthesized and its crystal structure determined.

In (I) (Fig. 1), the bond lengths and angles are in good agreement with the expected values (Allen *et al.*, 1987).

### Experimental

The title compound was synthesized by the reaction of 4-Chloro-phenylamine (1 mmol, 127.6 mg) with 3-Phenyl-propenal(1 mmol, 132.2 mg) in ethanol(20 ml) under reflux conditions (338 K) for 5 h. The solvent was removed and the solid product recrystallized from tetrahydrofuran. After five days yellow crystals suitable for X-ray diffraction study were obtained.

### Refinement

All H atoms were placed in idealized positions (C—H = 0.93Å) and refined as riding atoms with *U*_iso_(H) = 1.2*U*_eq_(C).

### Crystal data

**Table:**

C_15_H_12_ClN	*F*_000_ = 504
*M**_r_* = 241.71	*D*_x_ = 1.263 Mg m^−^^3^
Orthorhombic, *P**n**a*2_1_	Mo *K*α radiation λ = 0.71073 Å
Hall symbol: P 2c -2n	Cell parameters from 3328 reflections
*a* = 7.7333 (7) Å	θ = 2.7–27.2º
*b* = 5.5957 (5) Å	µ = 0.28 mm^−^^1^
*c* = 29.383 (3) Å	*T* = 295 (2) K
*V* = 1271.5 (2) Å^3^	Block, yellow
*Z* = 4	0.15 × 0.12 × 0.08 mm

### Data collection

**Table:**

Bruker APEXII CCD area-detector diffractometer	2187 independent reflections
Radiation source: fine-focus sealed tube	2042 reflections with *I* > 2σ(*I*)
Monochromator: graphite	*R*_int_ = 0.016
*T* = 295(2) K	θ_max_ = 25.0º
φ and ω scans	θ_min_ = 2.8º
Absorption correction: multi-scan(SADABS; Bruker, 2005)	*h* = −9→9
*T*_min_ = 0.960, *T*_max_ = 0.978	*k* = −6→4
6043 measured reflections	*l* = −32→35

### Refinement

**Table:**

Refinement on *F*^2^	Hydrogen site location: inferred from neighbouring sites
Least-squares matrix: full	H-atom parameters constrained
*R*[*F*^2^ > 2σ(*F*^2^)] = 0.026	*w* = 1/[σ^2^(*F*_o_^2^) + (0.0375*P*)^2^ + 0.0706*P*] where *P* = (*F*_o_^2^ + 2*F*_c_^2^)/3
*wR*(*F*^2^) = 0.066	(Δ/σ)_max_ = 0.001
*S* = 1.06	Δρ_max_ = 0.09 e Å^−^^3^
2187 reflections	Δρ_min_ = −0.15 e Å^−^^3^
154 parameters	Extinction correction: none
Primary atom site location: structure-invariant direct methods	Absolute structure: Flack (1983), 1031 Friedel pairs
Secondary atom site location: difference Fourier map	Flack parameter: 0.07 (5)

### Special details

**Table:**

Geometry. All e.s.d.'s (except the e.s.d. in the dihedral angle between two l.s. planes) are estimated using the full covariance matrix. The cell e.s.d.'s are taken into account individually in the estimation of e.s.d.'s in distances, angles and torsion angles; correlations between e.s.d.'s in cell parameters are only used when they are defined by crystal symmetry. An approximate (isotropic) treatment of cell e.s.d.'s is used for estimating e.s.d.'s involving l.s. planes.
Refinement. Refinement of *F*^2^ against ALL reflections. The weighted *R*-factor *wR* and goodness of fit *S* are based on *F*^2^, conventional *R*-factors *R* are based on *F*, with *F* set to zero for negative *F*^2^. The threshold expression of *F*^2^ > σ(*F*^2^) is used only for calculating *R*-factors(gt) *etc*. and is not relevant to the choice of reflections for refinement. *R*-factors based on *F*^2^ are statistically about twice as large as those based on *F*, and *R*- factors based on ALL data will be even larger.

### Fractional atomic coordinates and isotropic or equivalent isotropic displacement parameters (Å^2^)

**Table:**

	*x*	*y*	*z*	*U*_iso_*/*U*_eq_	
Cl1	0.65759 (7)	0.44278 (11)	0.969692 (18)	0.08065 (18)	
N1	0.87735 (18)	0.5317 (3)	0.77774 (5)	0.0522 (3)	
C1	0.7249 (2)	0.4739 (3)	0.91350 (6)	0.0509 (4)	
C2	0.8238 (2)	0.6663 (3)	0.90138 (5)	0.0535 (4)	
H2	0.8555	0.7795	0.9230	0.064*	
C3	0.8761 (2)	0.6907 (3)	0.85660 (5)	0.0503 (4)	
H3	0.9443	0.8203	0.8483	0.060*	
C4	0.82813 (19)	0.5241 (3)	0.82396 (6)	0.0430 (3)	
C5	0.7294 (2)	0.3300 (3)	0.83740 (6)	0.0483 (4)	
H5	0.6984	0.2152	0.8160	0.058*	
C6	0.6762 (2)	0.3037 (3)	0.88200 (6)	0.0524 (4)	
H6	0.6088	0.1737	0.8906	0.063*	
C7	0.9240 (2)	0.7278 (3)	0.75959 (6)	0.0499 (4)	
H7	0.9253	0.8657	0.7773	0.060*	
C8	0.9747 (2)	0.7431 (3)	0.71272 (5)	0.0477 (3)	
H8	0.9650	0.6067	0.6948	0.057*	
C9	1.0343 (2)	0.9406 (3)	0.69352 (5)	0.0504 (4)	
H9	1.0425	1.0744	0.7122	0.060*	
C10	1.0884 (2)	0.9711 (3)	0.64651 (5)	0.0421 (3)	
C11	1.1805 (2)	1.1742 (3)	0.63356 (5)	0.0493 (4)	
H11	1.2065	1.2901	0.6552	0.059*	
C12	1.2339 (2)	1.2059 (3)	0.58897 (6)	0.0541 (4)	
H12	1.2960	1.3419	0.5810	0.065*	
C13	1.1957 (2)	1.0385 (3)	0.55664 (6)	0.0531 (4)	
H13	1.2307	1.0608	0.5267	0.064*	
C14	1.1043 (2)	0.8352 (3)	0.56881 (5)	0.0505 (4)	
H14	1.0791	0.7201	0.5470	0.061*	
C15	1.0509 (2)	0.8028 (3)	0.61284 (5)	0.0452 (3)	
H15	0.9886	0.6664	0.6204	0.054*	

### Atomic displacement parameters (Å^2^)

**Table:**

	*U*^11^	*U*^22^	*U*^33^	*U*^12^	*U*^13^	*U*^23^
Cl1	0.0921 (4)	0.1047 (4)	0.0451 (2)	−0.0037 (3)	0.0100 (2)	0.0165 (3)
N1	0.0539 (8)	0.0588 (8)	0.0439 (8)	−0.0028 (6)	0.0035 (6)	0.0003 (6)
C1	0.0462 (9)	0.0647 (11)	0.0417 (8)	0.0070 (8)	0.0009 (7)	0.0096 (8)
C2	0.0597 (10)	0.0562 (10)	0.0448 (9)	−0.0048 (8)	−0.0048 (7)	−0.0015 (8)
C3	0.0522 (9)	0.0502 (9)	0.0487 (9)	−0.0114 (8)	−0.0012 (7)	0.0045 (8)
C4	0.0383 (7)	0.0467 (8)	0.0441 (8)	0.0020 (6)	−0.0021 (7)	0.0021 (7)
C5	0.0484 (9)	0.0431 (8)	0.0535 (9)	−0.0021 (7)	0.0010 (7)	−0.0011 (7)
C6	0.0496 (9)	0.0503 (9)	0.0573 (10)	−0.0071 (7)	0.0031 (8)	0.0092 (8)
C7	0.0480 (8)	0.0569 (9)	0.0448 (8)	0.0002 (7)	−0.0011 (7)	0.0019 (7)
C8	0.0464 (8)	0.0556 (9)	0.0412 (7)	−0.0002 (7)	−0.0005 (7)	−0.0001 (6)
C9	0.0534 (9)	0.0527 (9)	0.0450 (8)	−0.0019 (7)	−0.0028 (7)	−0.0021 (7)
C10	0.0392 (7)	0.0449 (8)	0.0423 (8)	0.0034 (6)	−0.0041 (6)	0.0052 (6)
C11	0.0499 (9)	0.0444 (8)	0.0537 (9)	−0.0028 (7)	−0.0055 (7)	0.0005 (7)
C12	0.0515 (10)	0.0483 (9)	0.0625 (11)	−0.0021 (7)	0.0037 (8)	0.0143 (8)
C13	0.0553 (10)	0.0565 (10)	0.0477 (10)	0.0064 (7)	0.0035 (7)	0.0099 (8)
C14	0.0584 (10)	0.0500 (9)	0.0432 (9)	0.0057 (7)	−0.0044 (7)	−0.0017 (7)
C15	0.0481 (8)	0.0426 (7)	0.0449 (8)	−0.0032 (7)	−0.0052 (7)	0.0041 (7)

### Geometric parameters (Å, °)

**Table:**

Cl1—C1	1.7399 (17)	C8—C9	1.324 (2)
N1—C7	1.273 (2)	C8—H8	0.9300
N1—C4	1.411 (2)	C9—C10	1.454 (2)
C1—C2	1.368 (2)	C9—H9	0.9300
C1—C6	1.380 (3)	C10—C11	1.394 (2)
C2—C3	1.383 (2)	C10—C15	1.397 (2)
C2—H2	0.9300	C11—C12	1.385 (2)
C3—C4	1.388 (2)	C11—H11	0.9300
C3—H3	0.9300	C12—C13	1.367 (3)
C4—C5	1.385 (2)	C12—H12	0.9300
C5—C6	1.382 (2)	C13—C14	1.386 (3)
C5—H5	0.9300	C13—H13	0.9300
C6—H6	0.9300	C14—C15	1.370 (2)
C7—C8	1.434 (2)	C14—H14	0.9300
C7—H7	0.9300	C15—H15	0.9300
			
C7—N1—C4	120.37 (14)	C9—C8—H8	118.2
C2—C1—C6	121.41 (16)	C7—C8—H8	118.2
C2—C1—Cl1	119.54 (14)	C8—C9—C10	127.14 (15)
C6—C1—Cl1	119.05 (14)	C8—C9—H9	116.4
C1—C2—C3	119.25 (16)	C10—C9—H9	116.4
C1—C2—H2	120.4	C11—C10—C15	117.55 (14)
C3—C2—H2	120.4	C11—C10—C9	120.17 (14)
C2—C3—C4	120.87 (15)	C15—C10—C9	122.27 (14)
C2—C3—H3	119.6	C12—C11—C10	121.00 (15)
C4—C3—H3	119.6	C12—C11—H11	119.5
C5—C4—C3	118.49 (15)	C10—C11—H11	119.5
C5—C4—N1	116.52 (14)	C13—C12—C11	120.35 (16)
C3—C4—N1	124.95 (14)	C13—C12—H12	119.8
C6—C5—C4	121.20 (15)	C11—C12—H12	119.8
C6—C5—H5	119.4	C12—C13—C14	119.57 (15)
C4—C5—H5	119.4	C12—C13—H13	120.2
C5—C6—C1	118.77 (15)	C14—C13—H13	120.2
C5—C6—H6	120.6	C15—C14—C13	120.39 (15)
C1—C6—H6	120.6	C15—C14—H14	119.8
N1—C7—C8	122.08 (15)	C13—C14—H14	119.8
N1—C7—H7	119.0	C14—C15—C10	121.14 (14)
C8—C7—H7	119.0	C14—C15—H15	119.4
C9—C8—C7	123.66 (15)	C10—C15—H15	119.4
			
C6—C1—C2—C3	0.0 (3)	N1—C7—C8—C9	−175.51 (17)
Cl1—C1—C2—C3	−179.67 (13)	C7—C8—C9—C10	179.94 (15)
C1—C2—C3—C4	0.6 (2)	C8—C9—C10—C11	−166.99 (16)
C2—C3—C4—C5	−1.3 (2)	C8—C9—C10—C15	13.3 (3)
C2—C3—C4—N1	−178.92 (16)	C15—C10—C11—C12	−0.6 (2)
C7—N1—C4—C5	160.20 (15)	C9—C10—C11—C12	179.69 (15)
C7—N1—C4—C3	−22.1 (2)	C10—C11—C12—C13	0.5 (3)
C3—C4—C5—C6	1.3 (2)	C11—C12—C13—C14	−0.6 (3)
N1—C4—C5—C6	179.18 (14)	C12—C13—C14—C15	0.7 (2)
C4—C5—C6—C1	−0.7 (2)	C13—C14—C15—C10	−0.7 (2)
C2—C1—C6—C5	0.1 (2)	C11—C10—C15—C14	0.7 (2)
Cl1—C1—C6—C5	179.73 (13)	C9—C10—C15—C14	−179.60 (15)
C4—N1—C7—C8	−179.89 (14)		
